# The Neural and Perceptual Effects of Stevia During Retronasal Occlusion

**DOI:** 10.1111/ejn.70469

**Published:** 2026-03-28

**Authors:** Hee‐kyoung Ko, Jingang Shi, Thomas Eidenberger, Weiyao Shi, Ciara McCabe

**Affiliations:** ^1^ School of Psychology and Clinical Language Sciences University of Reading Reading UK; ^2^ EPC Natural Products Co., Ltd. Beijing China; ^3^ University of Applied Sciences Upper Austria Wels Austria

**Keywords:** brain, neuroimaging, non‐nutritive sweeteners, smell, taste

## Abstract

We have recently shown that occluding retronasal pathways with a nose clip reduces both the subjective and neural responses to sucrose, suggesting the involvement of retronasal pathways in sucrose perception. However, how other sweet tastes such as stevia might also be affected by retronasal occlusion at the subjective and neural level is unknown. We examined the neural activity to stevia with a nose clip on (blocking retronasal pathways) and nose clip off, in a robust sample of healthy adults (*N* = 34, mean 25 years). Neural activity to stevia was reduced with the nose clip on in the olfactory cortex, hypothalamus, the subgenual and pregenual anterior cingulate and the nucleus accumbens. Stevia pleasantness was tracked by the posterior insula, but this was not apparent with the nose clip on. In conclusion, our findings are the first to demonstrate that blocking retronasal pathways significantly reduces neural responses to stevia taste, supporting the proposal that retronasal pathways play a role in the perception of tastes like stevia, and that stevia‐sweetened products could be made more palatable via retronasal pathways.

## Graphical abstract

Neural activity to stevia decreases with retronasal occlusion via nose clip. Insula tracks pleasantness but not with the nose clip on. In conclusion, blocking retronasal pathways reduces stevia taste responses, supporting their role in taste perception and enhancing stevia product palatability.

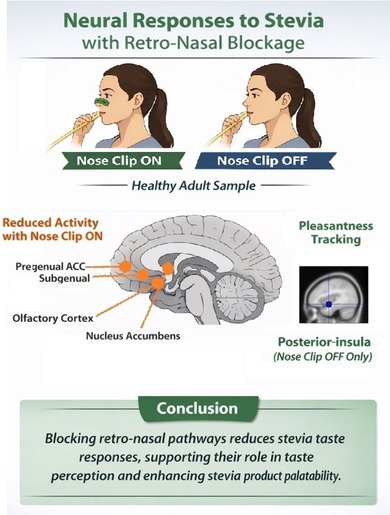

**Abbreviations**
EAT: Eating Attitude Test
FA: flip angle
FDR: false discovery rate
FOV: field of view
FWE: family‐wise error
mOFC: medial orbitofrontal cortex
NAcc: nucleus accumbens
NC0: nose clip off
NC1: nose clip on
OFC: orbitofrontal cortex
pgACC: pregenual cingulate
ROI: regions of interest
sgACC: subgenual anterior cingulate cortex
TE: echo time
TR: repetition time
WTH: waist‐to‐height ratio

## 1 Introduction

We are living in an obesity pandemic (WHO 2020) and food producers are encouraged to make foods low in calories. One way to do this is to use sweeteners such as stevia, a natural sweetener derived from the stevia plant native to South America that has been used as a sweetener for hundreds of years (Ashwell 2015). The high‐purity stevia leaf extract is being used globally to reduce energy intake and added sugar content in foods and beverages (Lemus‐Mondaca et al. 2012; Ashwell 2015; Rogers 2018; Stamataki, Crooks, et al. 2020; Stamataki, Scott, et al. 2020), and it does not negatively affect appetite (Zare et al. 2025). Stevia's main sweetening compounds are steviol glycosides such as stevioside and rebaudioside A and trace amounts of rebaudioside D and M (Koyama et al. 2003; Lemus‐Mondaca et al. 2012; Samuel et al. 2018). However, it is unclear how the neural response to stevia plays a role in its perception and if retronasal pathways are involved, as was recently shown for sucrose (He et al. 2024).

Retronasal pathways are activated by volatile odour molecules released in the mouth (aromas) that can travel up the back of the throat into the nasal cavity and activate the olfactory receptors (Stevens and Cain 1986; Voirol and Daget 1986; Buettner et al. 2001).

Previous studies show that disabling retronasal sensation through reversed nasal airflow or using a nose clip can significantly impair participants' ability to identify sucrose (Mozell et al. 1969; Murphy and Cain 1980; Masaoka et al. 2010), and its perceived sweetness intensity (Mojet et al. 2003; Mu et al. 2024; Yang et al. 2024) and mouth fullness (Baraniuk 2011; Yeomans and Boakes 2016) suggesting an important olfactory component (Yang et al. 2024). Consistent with this, our recent study found that blocking retronasal pathways via a nose clip reduced the subjective experience of mouth fullness and also neural activity during the taste of sucrose (Ko, Shi, Eidenbrger, et al. 2025). Specifically, we found reduced neural activity in the primary taste, olfactory, attention and reward regions of interest (ROI), and in the rolandic operculum, lingual gyrus and precuneus in the whole‐brain analyses. Further olfactory and prefrontal cortex ROIs tracked subjective mouth fullness, but this was not apparent with the nose clip on. However, there is very little data on the contribution of retronasal pathways to sweeteners such as stevia in the human brain. Hence, we hypothesised that blocking retronasal sensation could impact stevia taste perception and neural activity, similar to sucrose (Ko, Shi, Eidenbrger, et al. 2025).

The neural response to stevia has only been examined in a few studies. A previous study by Stamataki et al. (2022) examined the effects of caloric (glucose and maltodextrin) and noncaloric (stevia) sweet tastes on the human brain focusing on effects over time (up to 30 min postingestion). They confirmed that consumption of the caloric and noncaloric sweet drinks activated similar brain regions such as the prefrontal cortex, striatum, insula and cingulate cortex (Stamataki et al. 2022). They also found that stevia resulted in decreased activity over time in regions such as the motor, frontal areas and insula, similar to previous studies with glucose (Little et al. 2014). This finding could relate to stevia's prolonged sweet lingering effects (Lemus‐Mondaca et al. 2012). Stevia also has a slow onset of sweetness, bitterness at higher concentrations and has less mouth fullness compared to sucrose (Lavin et al. 2002; Lemus‐Mondaca et al. 2012). We recently also examined the neural response to stevia in a study on flavour modifiers and found that stevia activated similar brain regions to those in Stamataki et al.’s study and to the taste of sucrose (Ko, Shi, Eidenberger, et al. 2025b).

Therefore, in this study, the aim was to examine the neural and subjective response to the taste of stevia and how it might be affected by retronasal blockade via a nose clip. As sweet tastes are known to activate the insula (primary taste cortex), postcentral gyrus (somatosensory region), the hypothalamus and the orbitofrontal cortex (OFC; possibly secondary taste cortex), we identified these areas as regions of interest (Rolls 2019; Roberts et al. 2020; Yeung and Wong 2020). We also aimed to examine the amygdala as it responds to taste and oral texture (Kadohisa et al. 2005) and projects to olfactory regions, responding to both pleasant and unpleasant tastes (O'Doherty et al. 2001; Gottfried et al. 2003; Izadi and Radahmadi 2022), and the nucleus accumbens, implicated in the rewarding aspects of sweet tastes (Berridge 2009). Given our interest in retronasal pathways, we also aimed to examine the olfactory cortex, piriform cortex and the pregenual cingulate (pgACC) highlighted in a study examining neural differences to orthonasal vs. retronasal odour delivery (Small et al. 2005). Finally, we aimed to examine the subgenual anterior cingulate cortex (sgACC) as a region of interest as it has been shown involved in involuntarily attention to odours (Veldhuizen and Small 2011).

We also aimed to collect the subjective experiences of the taste of stevia in the scanner (pleasantness, bitterness and mouth fullness) so that we could correlate them with neural activity and examine the contribution of retronasal pathways to these processes.

## 2 Materials and Methods

### 2.1 Participants

Thirty‐four healthy, right‐handed adults (10 male and 24 female) were recruited for the fMRI study. All participants were between 18 and 45 years old and had a current body mass index (BMI, weight in kg/height in m^2^) or waist‐to‐height ratio (WTH) in the healthy range. Participants were excluded if they had any current/previous psychiatric history using the Structured Clinical Interview for DSM‐IV Axis I Disorder Schedule (SCID), or if they took psychoactive medication or had an eating disorder (measured with Eating Attitude Test [EAT] > 20), food allergies, diabetes, smoked or had any contraindications to fMRI scanning.

We also recorded the frequency, liking and craving for sugary and sweetened foods (Rolls and McCabe 2007). The questions in this scale consisted of ‘How frequently do you eat sugary foods?’, with answers of either a few times per month, one to two times per week, three to four times per week, or more than five times per week and ‘How frequently do you eat/drink foods with sweeteners?’, with answers of either never, rarely, sometimes, often, usually or always. The craving and liking for sugary foods were scored as 1 for low craving and 10 for high craving on a Likert scale.

All procedures contributing to this work comply with the ethical standards of the Helsinki Declaration of 1975, as revised in 2013, and ethical approval was obtained from the University of Reading Ethics committee, ethics ref.: 2023‐130‐CM; all participants provided written informed consent.

### 2.2 Demographic Data for fMRI Study

A total of 34 participants took part with a mean age of 25.71 years. See Table 1 for demographics.

**TABLE 1:** Demographics.

	All (*n* = 34), mean score (SD)
Age, years	25.71 (8.25)
Gender, female/male: *n*	24/10
Body mass index	22.00 (2.68)
EAT	3.09 (3.20)
Craving for sugary foods	5.11 (1.99)
Liking for sugary foods	5.85 (1.98)
Freq eating sugary foods	3.44 (2.09)
Freq eating/drinking foods with sweeteners	3.97 (2.11)

### 2.3 Pretest 1 (Triangle Test or Taste Perception Test)

The 34 participants were entered into the study if they could distinguish 2% sucrose from a control. This standard taste perception test was as follows: the participants were randomly allocated to the following sequences of two samples—A (distilled water) and B (20 g sucrose/L [2% sucrose]): ABB, AAB, ABA, BBA, BAA and BAB. For the individual performance, each participant received all six sequences in random order. In a sequence, the participants took the whole 10 mL of each sample into their mouth, swirled and coated the solution around their mouth for 3 s and then spit it into a spittoon. On each trial after tasting all three, they indicated which was different from the other two. If after all six trials participants did not get six out of six trials correct, they could have a second attempt as distinguishing distilled water from sucrose in distilled water is difficult and 100% accuracy is not expected in these types of tests, first time. Participants who yielded correct identification of at least five out of the six trials on a second attempt were also recruited to the study as this meant all participants would be correct above 80%. Twenty‐one participants passed the pretest with six out of six trials correct the first time (100%), a further 10 participants passed the pretest with five out of six trials correct the first time (83%). Only three participants had a second attempt but they then got six of the six trials correct (100%), satisfying the criteria of being able to identify sweet taste from distilled water above change levels (33%) (Bi 2008), and thus were also included in the study; this is also similar to the rates in previous studies (Ko, Shi, Eidenberger, et al. 2025a).

### 2.4 Pretest 2 (Candy Smell Test)

We used the candy smell test to check participants' retronasal olfactory performance (Renner et al. 2009). This test examines participants' ability to identify the flavour of a candy (500 mg) placed on the middle of the tongue from six possible choices (six‐alternative, forced‐choice procedure): strawberry, banana, orange, coffee, cherry or pineapple. Participants can suck the candy or chew it if necessary. The participants wrote down one of the choices. If they could not identify the candy, they could skip to the next trial. Each participant performed five trials with the nose clip on and five trials with the nose clip off. Between trials, participants rinsed their mouths with water. There was no feedback to the participants about whether their responses were correct or incorrect. We expected 80%–100% correct (four or five correct/five trials) for the nose clip off condition and less than 40%–50% correct (one or two correct/five trials) for the nose clip on condition in line with previous studies (Renner et al. 2009). As expected, we found that with the nose clip off, participants could identify the flavours in the candy smell test with average accuracy of 84% (±14), and when the nose clip was added, this accuracy dropped to 31% (±20) in line with previous studies (Renner et al. 2009). A paired‐samples *t*‐test confirmed this difference (*t*(33) = 13.35, *p* < 0.001).

### 2.5 Pretest (Smell Test Orthonasal)

To check participants' orthonasal olfactory performance, and to exclude anosmia, we used the coffee smell test (Humphries and Singh 2018). This is reported to have excellent validity with a sensitivity of 93% and a specificity of 96% in comparison to a 12‐item Sniffin Sticks test kit (Singh et al. 2018). We prepared a 100‐mL cup with grounded coffee beans and one empty cup. In a trial, the participants were asked to close their eyes and sniff from a cup that was presented to them blind (either coffee or empty); they had to report the smell by marking on 0–10 scales the smell intensity—0 indicated no smell at all, with 10 indicating a very strong smell. Each participant performed five trials with nose clip on and five trials with nose clip off. As expected, all participants could identify the cup that had coffee in compared to no coffee and rated the coffee smell as above average intensity. Participants rated the coffee smell (6.70 ± 1.78) higher than the intensity of the empty cup smell (1.17 ± 1.90), (*t*(22) = 12.04, *p* < 0.001) and rated the intensity of the coffee smell (6.70 ± 1.78) higher with the nose clip off than with the nose clip on (0.26 ± 0.59), (*t*(22) = 16.7, *p* < 0.001).

### 2.6 Stimuli for the Scan

For the fMRI scan, stevia was the basic stimulus. For the stevia used in this study, the detailed composition of steviol glycosides was analytically determined as follows: rebaudioside A (97.91%), stevioside (0.03%), rebaudioside F (0.42%), rebaudioside C (0.12%), rebaudioside B (0.24%) and other minor rebaudiosides (1.23%). The total steviol glycoside content therefore amounted to 99.95%. In addition, the ash content was measured at 0.01%, resulting in a total quantified and analysed material content of 99.96% and provided by EPC Natural Products Co., Ltd. (see supplementary information for more details). The concentration of stevia was 0.036%, a concentration chosen to be equivalent to that of 6% sucrose used in our previous study (Wee et al. 2018; Ko, Shi, Eidenberger, et al. 2025a). Stevia was diluted and delivered in distilled water. A tasteless solution (containing the main ionic components of saliva, 25 mM KCl + 2.5 mM NaHCO_3_) was used as a control rinse condition at the end of each trial.

### 2.7 Nose Clips

Soft plastic foam nose clips were used to block retronasal smell (size approx. 6.8 × 4 cm/2.7 × 1.6 in. in length and width) and sourced from Frienda Ltd., China. The pleasantness, pain and comfort of the nose clips were piloted before the study on eight subjects. All participants rated the nose clip on the nose between ‐4 and 4 for pleasure, pain and comfort, once at baseline and again after wearing the nose clip for 5 min (the length of time they would be wearing the nose clip in each condition in the scanner).

To examine the effects of the nose clip on subjective ratings, we used a repeated measures ANOVA with ratings (three levels: pleasantness, pain and comfort) as one within‐subject factor and time (two levels: time 1 and time 2) as a second within‐subject factor. We found no main effect of ratings (*F* = 0.165 (2,14) *p* = 0.85) or time (*F* = 0.1 (1,7) *p* = 0.75) and no ratings * time interaction (*F* = 2 (2,14) *p* = 0.17; Table 2).

**TABLE 2:** Nose clip test.

T1			T2		
Mean (SD)			Mean (SD)		
Pleas	Pain	Comfort	Pleas	Pain	Comfort
−0.16	−0.76	−0.26	−0.21	−0.42	−0.78
(1.09)	(1.90)	(1.51)	(1.41)	(1.81)	(1.69)

### 2.8 Study Design

The fMRI scans took place at the Centre of Integrative Neuroscience and Neurodynamics (CINN) at the University of Reading. If scheduled for a morning scan, participants fasted overnight; if having an afternoon scan, participants fasted for 3 h (no food, only water) before the scan. A total of 10 participants had a morning scan, and 24 participants had an afternoon scan. Before scanning (60–90 min), all the participants were given a standardised meal similar to previous studies (a banana, a cup of orange juice, two crackers, ~261 total calories) with the instruction to ‘eat until feeling comfortably full, without overeating’ similar to our previous study (Thomas et al. 2015; Ko, Shi, Eidenberger, et al. 2025a). We asked participants to rate their hunger and mood before the scan on a visual analogue scale from 0 being not at all to 10 indicating the most ever felt. Subjects were screened for potential pregnancy and metal in their body before being placed in the fMRI scanner.

#### 2.8.1 Taste Delivery

Tastes were delivered to the subject via separate long (~3 m) thin Teflon tubes with a mouthpiece (~1 cm in diameter) at one end, which was held by the subject comfortably between the centre of the lips. At the other end, the tubes were connected to separate reservoirs via syringes and one‐way Syringe Activated Dual Check Valves (Model 14044‐5, World Precision Instruments, Inc.), which allowed any stimulus to be delivered manually by the researcher at exactly the right time indicated by the programme (Murray et al. 2014), thus avoiding the delays and technical issues experienced when using computerised syringe drivers.

#### 2.8.2 fMRI Task

At the beginning of a trial, a white cross at the centre of the screen appeared for 2 s indicating the start. Then, stevia was delivered in a 0.5‐mL aliquot to the subject's mouth, the green cross was presented at the same time on the visual display for 5 s. The instruction given to the subject was to move the tongue once as soon as a stimulus was delivered in order to distribute the solution round the mouth to activate receptors, and then to keep still until a red cross was shown, when the subject could swallow. Swallowing was 2 s, then the subject was asked to rate the ‘pleasantness’ (−2 to +2) hedonic value, asked to rate the ‘bitterness’ (0 to +4), and asked to rate the ‘mouth fullness’ (richness) sensory aspect (0 to +4) of the taste in their mouth on a visual analogue scale by moving a bar to the appropriate point using a button box, similar to previous taste/fMRI studies (Rolls 2019). Each rating period was 5 s long. After the last rating on each trial, 0.5 mL tasteless control solution was administered in the same way as the stevia stimulus and a green cross was again presented at the same time on the visual display for 5 s. The control was used as the comparison condition to allow somatosensory effects produced by liquid in the mouth, and the single tongue movement made to distribute the liquid throughout the mouth, to be subtracted in the fMRI data analysis (O'Doherty et al. 2001; de Araujo et al. 2003). The tasteless control condition was not subjectively rated. A grey cross was presented for a duration between 0.8 and 2 s (jittered) to indicate the end of the trial. Then, the screen was black for 2 s before a new trial started. Each trial lasted ~30 s.

Using a block design, there were seven trials of stevia taste with nose clip on followed by a second block of seven trials of stevia taste with nose clip off, block order was counterbalanced across participants. Between blocks, the scanner was also stopped for ~5 to 10 min to allow the participant to either place the nose clip on or take it off, depending on the order of blocks. During the break, participants were told they could let go of the taste tubes and just relax and they could close their eyes. Stopping the scanner between nose clip addition or removal also allowed for movement to be minimised as the second block began with a new localiser scan. The whole task took ~30 min, including stopping and starting the scanner.

#### 2.8.3 fMRI Data Acquisition

Blood oxygenation level–dependent (BOLD) functional MRI images were acquired using a three‐Tesla Siemens scanner (Siemens AG, Erlangen, Germany) with a 32‐channel head coil. During the task, approximately 1500 volumes were obtained for each participant, using a multiband sequence with GRAPPA and an acceleration factor of 6. Other sequence parameters included a repetition time (TR) of 700 ms, an echo time (TE) of 30 ms and a flip angle (FA) of 90°. The field of view (FOV) covered the whole brain with a voxel resolution of 2.4 × 2.4 × 2.4 mm^3^. Moreover, structural T1‐weighted images were acquired utilising a magnetisation prepared rapid acquisition gradient echo sequence (TR = 2020 ms, TE = 3.02 ms, FA = 9°) with an FOV covering the whole brain and a voxel resolution of 1 × 1 × 1 mm^3^.

#### 2.8.4 fMRI Data Analysis

The imaging data were analysed using SPM12 (Wellcome Centre for Human Neuroimaging, University College London). Preprocessing of the data used SPM12 realignment, coregister, segment, normalisation to the MNI coordinate system (Montreal Neurological Institute) and spatial smoothing with a 6‐mm full‐width at half‐maximum isotropic Gaussian kernel. The time series at each voxel was low‐pass filtered with a haemodynamic response kernel. Time series nonsphericity at each voxel was estimated and corrected for, with a high‐pass filter with a cut‐off period of 128 s.

In the single‐event design, a general linear model was then applied to the time course of activation in which stimulus onsets were modelled as single impulse response functions and then convolved with the canonical haemodynamic response function. Linear contrasts were defined to test specific effects. Time derivatives were included in the basis functions set. Following smoothness estimation, linear contrasts of parameter estimates were defined to test the specific effects of each condition with each individual dataset. Voxel values for each contrast resulted in a statistical parametric map of the corresponding *t* statistic (transformed into the unit normal distribution [SPM *z*]). Movement parameters were added as additional regressors.

At the second level, we examined the main effects of stevia with nose clip off vs. the corresponding control tasteless conditions with nose clip off, and stevia with nose clip on vs. stevia with nose clip off, thresholded at *p* < 0.05 corrected (family‐wise error [FWE] and *p* values cluster‐corrected at both *p* < 0.05 false discovery rate (FDR) and *p* < 0.05 FWE). We also added gender, hunger level and scan time as covariates of no interest.

We then examined regions of interest (ROI) spheres (10 mm) for the anterior insula (primary taste cortex, [−32, 16, 2]), posterior insula [−38, −2, −12] and postcentral gyrus [60, −16, 24] using WFU PickAtlas, and the hypothalamus using AAL atlas and identified from meta‐analyses on sweet tastes in humans (Roberts et al. 2020; Yeung and Wong 2020). We examined the olfactory regions; the piriform cortex, olfactory cortex and the orbitofrontal cortex using AAL atlas anatomical masks in WFU PickAtlas. Given our interest in retronasal effects (Small et al. 2005) and attention to odours (Veldhuizen and Small 2011), we also created a sphere (10 mm) in the pgACC [3, 42, −9] (Small et al. 2005) and examined anatomical masks of the medial orbitofrontal cortex (mOFC) (Small et al. 2005) and sgACC (BA25) (Veldhuizen and Small 2011) using AAL atlas in WFU PickAtlas. Finally, as we are interested in the retronasal contribution to the rewarding/aversive effects of stevia, we also examined the nucleus accumbens (Berridge 2009) and amygdala (Gottfried et al. 2003; Izadi and Radahmadi 2022) using IBASPM71 atlas and the AAL atlas anatomical masks, respectively, in WFU PickAtlas (Table 4). For the ROI analyses, data were extracted using the SPM ROI analysis Matlab code and SPM's spm_get_data command and analysed with paired‐sample *t* tests, in Excel and SPSS, and then corrected for multiple comparisons across the 18 ROIs, i.e., *p* = 0.05/18 = 0.003. We also examined correlations between the extracted ROI data and the subjective ratings of pleasantness, bitterness and mouth fullness.

## 3 Results

### 3.1 fMRI Scan Day

#### 3.1.1 Subjective Hunger and Mood

Participants rated their positive affect, alertness and happiness relatively higher than they rated the negative affect, e.g., sadness and disgust. Hunger levels were rated in the middle, suggesting participants felt neither too hungry nor too full (Table 3).

**TABLE 3:** Hunger and mood ratings.

Visual analogue scale	Mean score (±SD)
**Appetite**	
How hungry do you feel right now?	4.35 ± 2.30
How full do you feel right now?	4.05 ± 2.11
**Mood**	
Alertness	6.08 ± 2.40
Disgust	0.91 ± 1.23
Drowsiness	3.05 ± 2.66
Anxiety	1.79 ± 1.55
Happiness	6.11 ± 1.93
Nausea	0.70 ± 0.97
Sadness	0.55 ± 1.05
Withdrawn	1.08 ± 1.76
Faint	1.08 ± 1.84

*Note:* Rate between 0 and 10, where 0 = not at all, 10 = most ever felt.

#### 3.1.2 Subjective Ratings of Stevia During the Scan With Nose Clip On and Off

**FIGURE 1:**
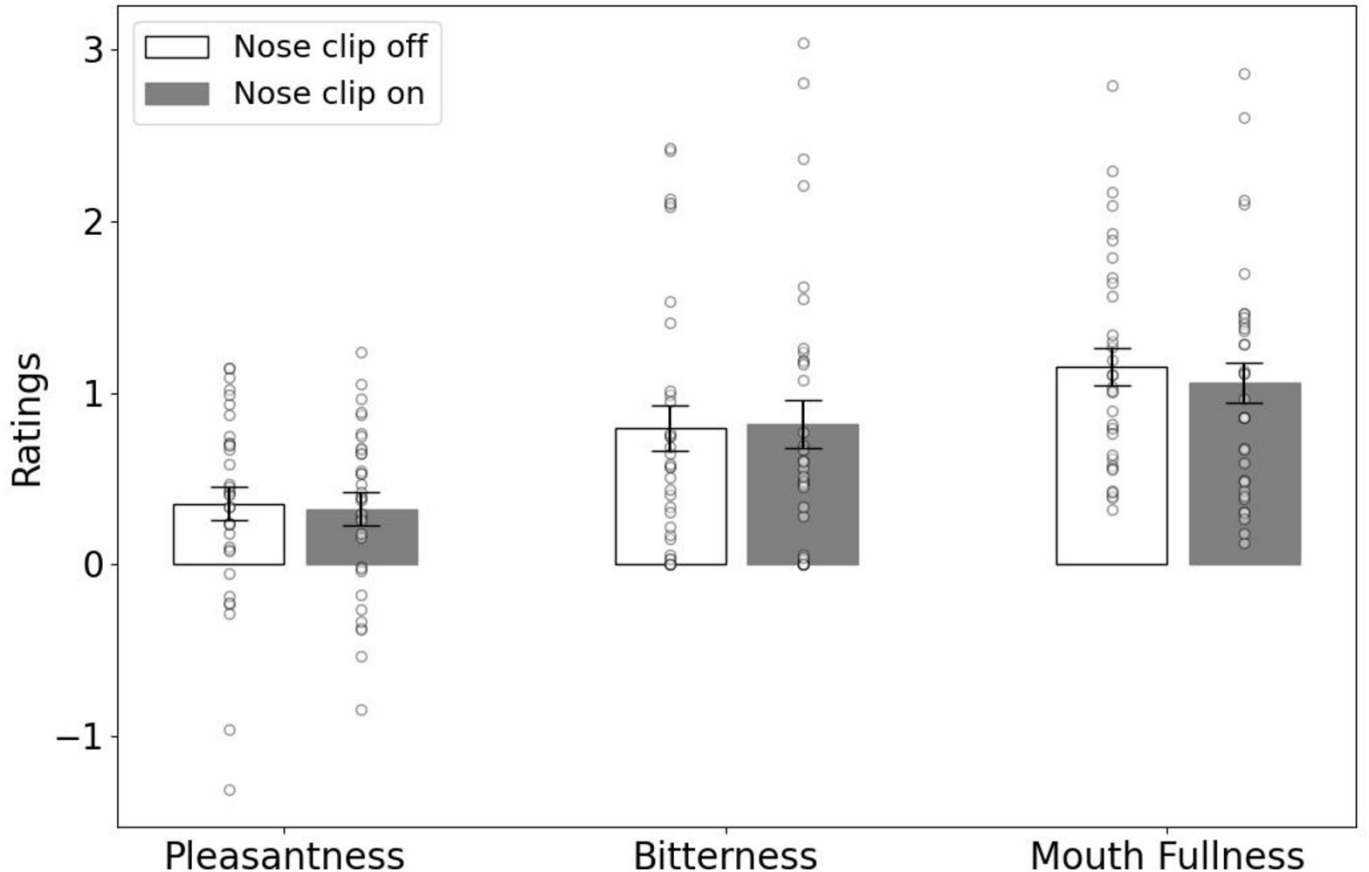
Pleasantness, bitterness and mouth fullness ratings for stevia with nose clip on (NC1) and nose clip off (NC0).

To examine the effects of the nose clip on subjective ratings, we used a repeated measures ANOVA with ratings made during the scans as a within factor (three levels: pleasantness, bitterness, mouth fullness) and condition (two levels: nose clip on, nose clip off) as a second within‐subject factor. We found a main effect of ratings (*F* = 17.2 (1.5, 50) *p* < 0.001), but no main effect of condition (*F* = 0.9 (1,33) *p* = 0.34), or ratings * condition interaction (*F* = 0.594 (1.69,56) *p* = 0.53; Figure 1).

### 3.2 Whole‐Brain Analysis

#### 3.2.1 Main Effects of Taste Stimuli

To examine if the task worked and the taste was activating parts of the brain expected, we used whole‐brain analyses to investigate the effect of stevia vs. the control taste. This revealed that, as expected, the stevia taste activated the primary taste cortex (insula), primary somatosensory cortex (postcentral gyrus), precentral gyrus, caudate and putamen, similar to previous studies on sweet tastes (Yeung and Wong 2020) (Table S1). Next, we examined the opposite contrast to see if there was more activation to the control taste solution vs. stevia and found that there were no significant increased activations for the control vs. stevia contrast.

### 3.3 ROI Analysis

#### 3.3.1 Stevia: Nose Clip Off vs. Nose Clip On

**FIGURE 2:**
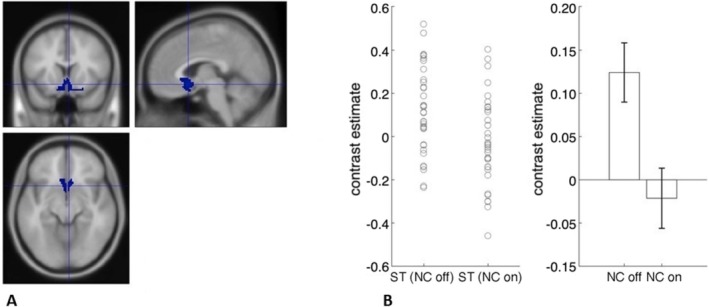
A, Olfactory ROI. B, Contrast estimates extracted from ROI using MarsBaR for stevia nose clip off and nose clip on (error bars, SEM).

**FIGURE 3:**
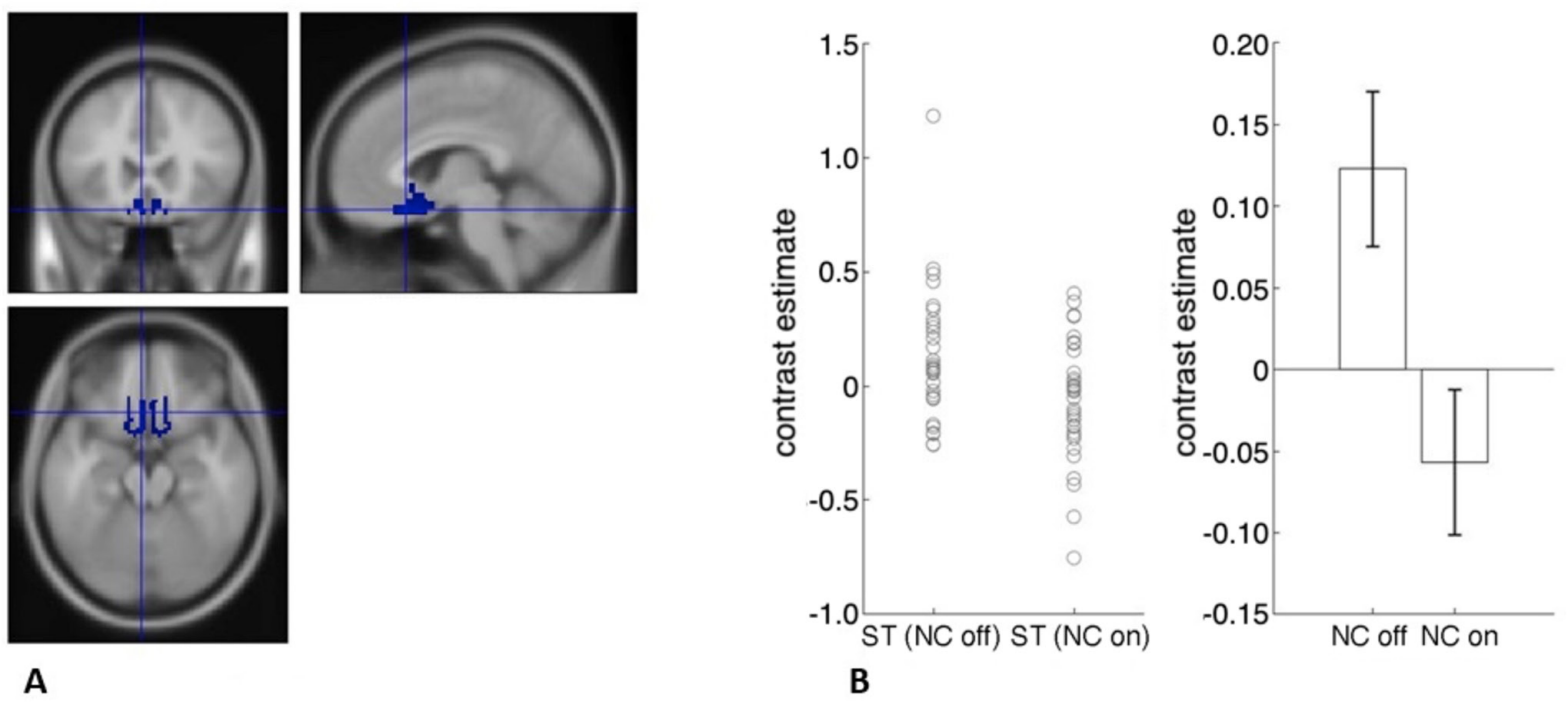
A, sgACC (BA 25) ROI. B, Contrast estimates extracted from ROI using MarsBaR for stevia nose clip off and nose clip on (error bars, SEM).

**FIGURE 4:**
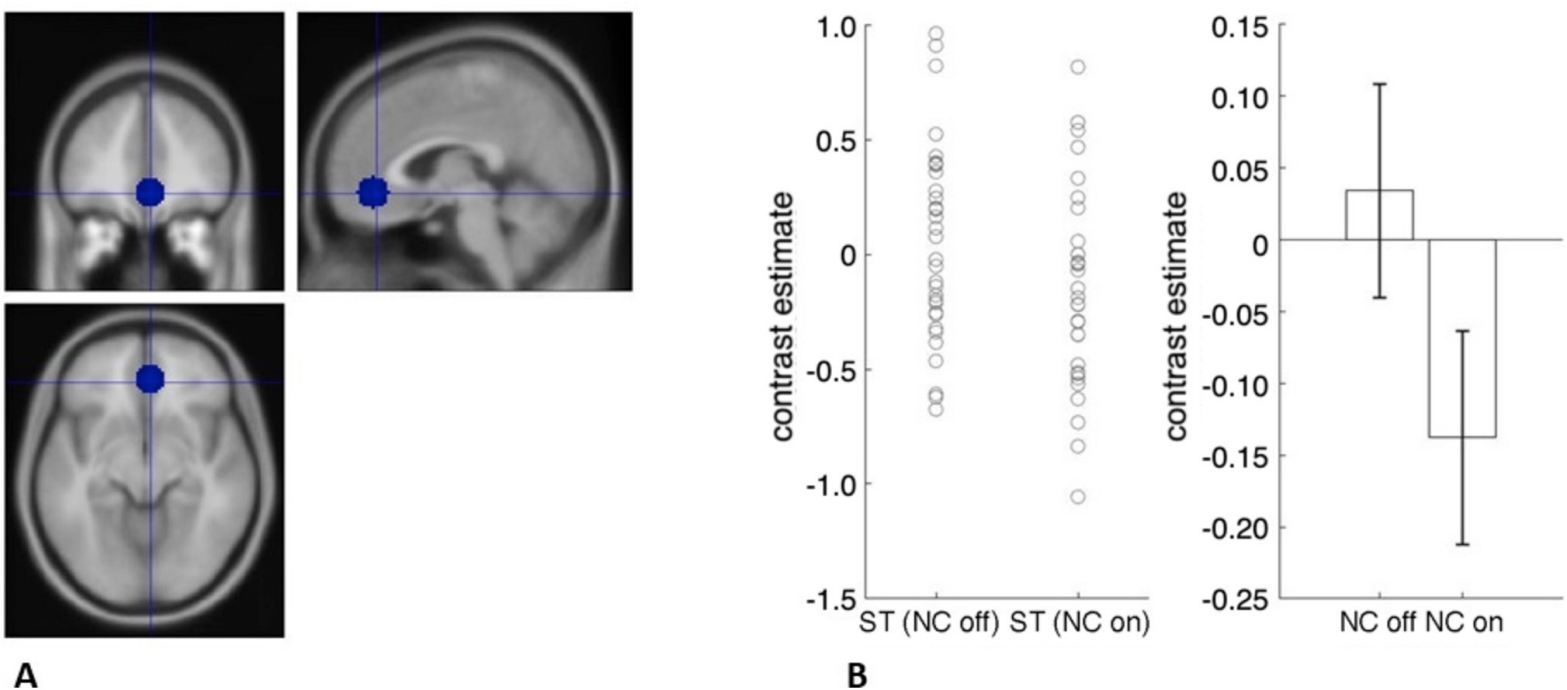
A, pgACC ROI. B, Contrast estimates extracted from ROI using MarsBaR for stevia nose clip off and nose clip on (error bars, SEM).

Next, we examined with ROI analyses the main hypotheses that blocking the retronasal pathways with a nose clip would blunt the neural response to stevia. We did this by comparing brain activity to stevia with the nose clip off vs. stevia with the nose clip on. We also did this for each ROI. We found activity in the olfactory cortex, sgACC and pgACC, hypothalamus and right NAcc reduced with the retronasal pathways occluded with a nose clip. We also examined the results after controlling for multiple comparisons, i.e., the number of ROIs examined (Table 4, Figures 2, 3, 4). Next, we examined if there were brain regions that had greater neural activity for the opposite contrast of nose clip on vs. off.

**TABLE 4:** Effect of nose clip in ROIs.

Stevia nose clip off vs nose clip on						
ROI	***t*** value	***p***	Cohens ***D***			
Olfactory	4.16	0.0001*	0.71			
Piriform	1.63	0.06	0.28			
sgACC	3.43	0.0008*	0.59			
mOFC	1.76	0.04	0.30			
pgACC	3.03	0.002*	0.52			
Hypothalamus	3.34	0.001*	0.57			

	Left			Right		
	***t*** value	***p*** value	Cohens ***D***	***t*** value	***p*** value	Cohens ***D***
Postcentral gyrus	2.29	0.01	0.39	1.64	0.05	0.28
Anterior insula	2.81	0.004	0.48	2.05	0.02	0.35
Posterior insula	0.56	0.29	0.10	1.47	0.08	0.25
NAcc	2.82	0.004	0.52	2.91	0.003*	0.51
OFC	2.3	0.014	0.39	0.15	0.44	0.03
Amygdala	1.89	0.03	0.32	2.35	0.01	0.40

* Survives correction for multiple comparisons (0.05/18 ROIs, *p* = 0.003).

### 3.4 Parametric Modulation

Next, we examined the relationships between the subjective ratings of stevia and the neural activity for each of the ROIs. We did this for the nose clip off condition and then the nose clip on condition. This was to see if there were activations in the brain tracking the subjective experience of stevia (pleasantness, bitterness, mouth fullness) and if these activations were then blunted by the addition of the nose clip (retronasal blockade).

#### 3.4.1 Pleasantness

**FIGURE 5:**
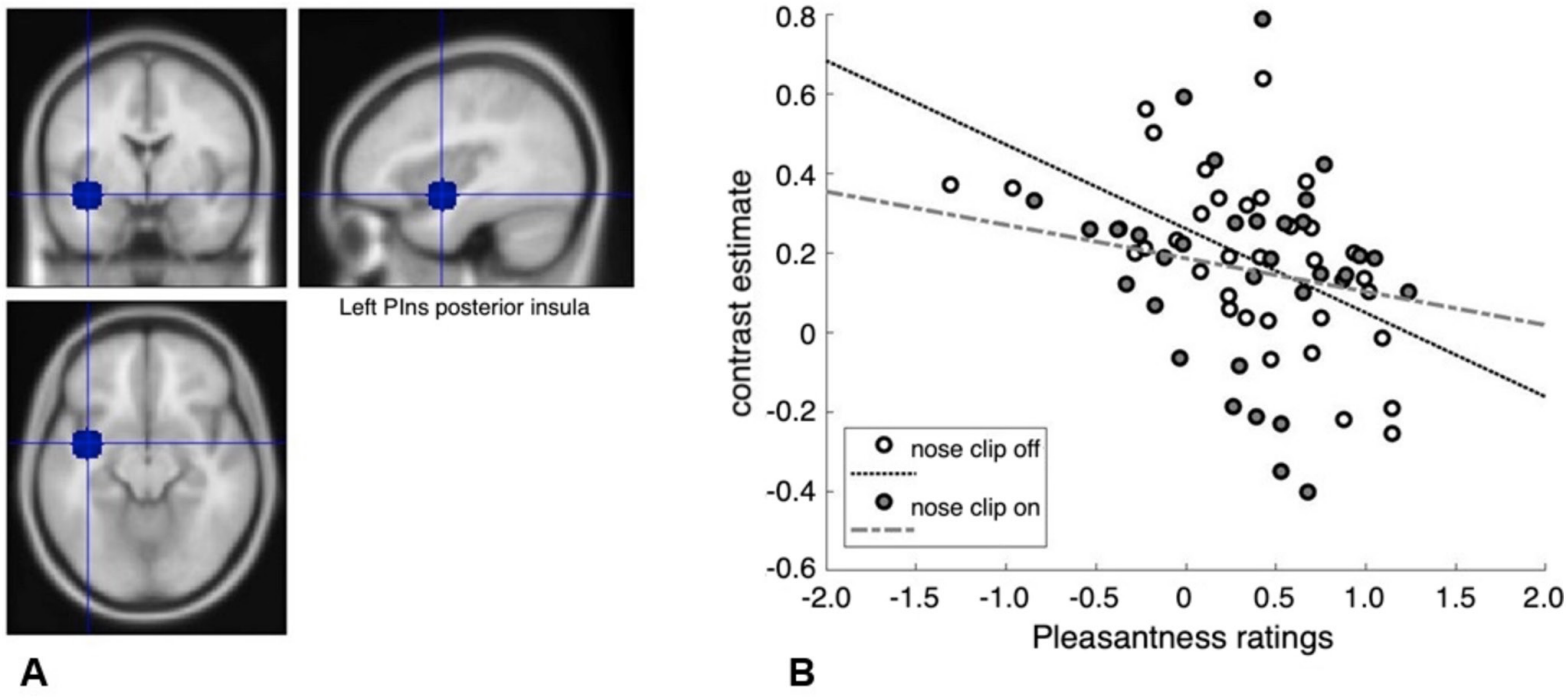
A, Posterior insula ROI. B, Correlations between stevia pleasantness ratings and contrast estimates extracted from ROI using MarsBaR.

We found a negative correlation between stevia pleasantness and ROI activity in the posterior insula (left: *rho* = −0.56, *p* = 0.0006, two‐tailed) for nose clip off, which survived correction for multiple comparisons but not for nose clip on (left: *rho* = −0.169, *p* = 0.33, two‐tailed). To directly compare the correlations, we used the Fishers *z* transform and found they were significantly different (*z* = −1.82, *p* = 0.03; Figure 5).

### 3.5 Exploratory Whole‐Brain Analysis

When examining the effects of the stevia nose clip on vs. off and nose clip off vs. on, there were no effects in the whole brain.

## 4 Discussion

Stevia activated brain regions similar to those activated by other sweet tastes such as sucrose reported in previous studies (Roberts et al. 2020; Yeung and Wong 2020; Stamataki et al. 2022; Ko, Shi, Eidenberger, et al. 2025a, 2025b; Ko, Shi, Eidenbrger, et al. 2025). We also provide novel first evidence that retronasal occlusion with a nose clip reduces neural activity to stevia in the olfactory cortex, hypothalamus, sgACC, pgACC and nucleus accumbens.

The reduced activity with the nose clip on in areas related to taste, odour and reward processing (olfactory, hypothalamus, sgACC, pgACC, NAcc) demonstrates a potential biological mechanism for the reduced subjective experience of stevia with retronasal blockade (He et al. 2024) and provides further support for the notion of retronasal pathways as contributors to the perceptual processing of sweeteners such as stevia (He et al. 2023; He et al. 2024; Ko, Shi, Eidenbrger, et al. 2025). Consistent with our findings, previous work by Small et al. (2005) also found greater pgACC activity to retronasal vs. orthonasal smell delivery.

Reduced olfactory and hypothalamic activity with the nose clip on suggests that when tasting stevia, the retronasal pathways are activating regions involved in smell, appetite and metabolic responses. The olfactory cortex receives projections from retronasal pathways, including the piriform cortex and orbitofrontal cortex, integrates this retronasal information with other sensory inputs like taste and texture (Shepherd 2007) and was found reduced in activity with retronasal blockade during sucrose tasting (Ko, Shi, Eidenbrger, et al. 2025).

The hypothalamus has been implicated in appetite and metabolic processes, and studies also report its involvement in taste perception (Pu et al. 2024); therefore, reduced hypothalamus with nose clip on during stevia taste could indicate a reduced ability to fully interpret stevia.

Reduced anterior cingulate and nucleus accumbens activity to stevia with the nose clip on is consistent with our previous study on retronasal blockade for sucrose taste (Ko, Shi, Eidenbrger, et al. 2025) and indicates retronasal pathways are activating regions involved in emotion, reward and decision‐making. Furthermore, previous studies show that prefrontal cortex regions such as the pgACC are multimodal regions integrating taste and smell information (de Araujo et al. 2003), and show greater activation to tastes when combined with odours than to the sum of the activations by the taste and olfactory components presented separately (McCabe and Rolls 2007; Rolls 2019). This could suggest that the nose clip reduces the integration of taste and olfactory components, making it more difficult to perceive stevia. Furthermore, as studies find that odour/preference learning in rats is more effective from retronasal vs. orthonasal routes (Blankenship et al. 2019), our findings could indicate that blocking retronasal processing of stevia with a nose clip could alter decision‐making in relation to stevia. Future studies using a fMRI decision‐making task could therefore examine if retronasal blockade with a nose clip would disrupt neural responses during decision‐making and hence interrupt subsequent food choice behaviour, further implicating a role for retronasal pathways.

We also found reduced activity in the NAcc, a hub related to feeding, homeostatic and hedonic circuits, that facilitates behaviour via its downstream projections (Marinescu and Labouesse 2024), when examining the effects of retronasal blockade, similar to our previous study on retronasal blockade during sucrose tasting (Ko, Shi, Eidenbrger, et al. 2025). The ventral striatum is at the crossroads of olfactory and reward pathways and receives direct projections from the primary olfactory cortex (Ubeda‐Bañon et al. 2007) and the dopaminergic midbrain (Ikemoto 2007), and is greatly involved in odour‐guided eating behaviour (Murata 2020). Therefore, reduced activity in the NAcc supports the idea that retronasal olfactory signals related to stevia are being occluded.

We also found that correlations between the subjective ratings (pleasantness) and the neural activity (insula) were significant with the nose clip off and nonsignificant with the nose clip on, and that these correlations were significantly different from each other. This further supports our proposal that retronasal pathways contribute to the neural processing of stevia (He et al. 2023; He et al. 2024), but at an unconscious level, as we found no significant difference between nose clip on vs. off when examining the subjective reports alone. These findings therefore highlight the power of neuroimaging to detect objective biological sensory effects, outside of subjective conscious awareness. Future studies should test if the regions that correlated with the subjective report can be used to predict subsequent choice behaviour in relation to stevia and if these predictions are affected by a nose clip.

Regards power, there seems to be little agreement on how to estimate statistical power for fMRI studies that rely on a very large number of tests and idiosyncratic statistical procedures (Poldrack et al. 2017). A recent meta‐analysis on how the brain processes sugars and sweeteners (Yeung and Wong 2020) finds only one previous study that did a power analysis to determine sample size (Van Opstal et al. 2021), which recruited *N* = 20 based on a power analysis showing an ~2% change in BOLD after glucose ingestion (Van Opstal et al. 2019). However, the authors still concluded that they were likely underpowered. Hence, we aimed to recruit a larger sample in line with guidance on fMRI power (Mumford 2012; Szucs and Ioannidis 2020). As within‐subject designs are more powerful than between‐subject designs, and a single one‐sample *t*‐test (two‐tailed) shows 34 participants will surpass 80% power to detect an effect size of *D*  =  0.5 at *α*  =  0.05 (Szucs and Ioannidis 2020), we recruited this number. As we know *p* values are not enough in fMRI reporting, we also give the effect sizes and some of these are much lower than 0.5. This is likely because studies involving sensory stimuli like taste (tongue/motor) can have some of the largest neural effects (Poldrack et al. 2017). We also employed ROI analyses decided upon before data collection and used strict corrected statistics for multiple comparisons; we report both the ROI data before and after multiple correction to allow readers to see areas of interest that would otherwise be excluded by our very strict corrections (0.05/18) aiding authors to reproduce our results.

In summary, we have shown with neuroimaging that retronasal pathways may be playing a role in the neural processing of stevia and contributing to its perceptual properties. In conclusion, this study contributes to a broader understanding of how retronasal pathways contribute to the neural processing of natural, zero‐calorie sweeteners such as stevia.

## Author Contributions

**Hee‐kyoung Ko:** methodology (equal), formal analysis (lead), writing – review and editing (equal). **Jingang Shi:** conceptualization (equal), methodology (equal), review and editing (equal). **Thomas Eidenberger:** methodology (equal), review and editing (equal). **Weiyao Shi:** methodology (equal), review and editing (equal). **Ciara McCabe:** conceptualization (equal), methodology (equal), writing – review and editing (equal).

## Funding

This work was supported by EPC Natural Products Co., Ltd.

## Conflicts of Interest

The authors declare the following financial interests/personal relationships, which may be considered as potential competing interests: Weiyao Shi and Jingang Shi are employees of EPC Natural Products Co., Ltd., who provided the compounds and funded the study. The work was conducted independently at the NRG laboratory of Prof. McCabe at the University of Reading, solely for the purpose of scientific understanding. All authors declare that they have no other known competing financial interests or personal relationships that could have appeared to influence the findings reported in this paper.

## Supporting information




**Table S1:** Stevia—control (nose clip off).
**Table S2:** Stevia—control (nose clip on).

## Data Availability

The data will be made available in a public repository.
