# Supplementary material for: The Neural and Perceptual Effects of Stevia During Retronasal Occlusion
Source: Eur J Neurosci. 2026 Mar 28;63(7):e70469. doi: 10.1111/ejn.70469 (PMC13032186; doi:10.1111/ejn.70469)
Supplement: Supplementary file 1 — Table S1: Stevia—control (nose clip off). Table S2: Stevia—control (nose clip on). [file EJN-63-0-s001.docx]

*Supplementary doc*

***The Neural and Perceptual Effects of Stevia during Retro Nasal Occlusion***

**Stevia composition**

For the stevia used in this study the detailed composition of steviol glycosides was analytically determined as follows: Rebaudioside A (97.91%), Stevioside (0.03%), Rebaudioside F (0.42%), Rebaudioside C (0.12%), Rebaudioside B (0.24%), and other minor rebaudiosides (1.23%). The total steviol glycoside content therefore amounted to 99.95%. In addition, the ash content was measured at 0.01%, resulting in a total quantified and analyzed material content of 99.96%.

GC–MS analysis was performed to screen for potential volatile compounds. Four trace-level volatiles were detected including benzaldehyde, limonene, nonanal, and decanal, each at concentrations below 0.6 ppm in the sample. Under the diluted experimental conditions employed in this study, the concentrations of volatile compounds were extremely low. Specifically, a 0.036% stevia solution (equivalent to 0.36 g Reb-A 97% per litre) contains less than 0.216 µg of volatiles per litre, corresponding to approximately 0.216 ng/mL of solution. At such trace levels, the contribution of these volatiles to sensory and perceptual outcomes can be considered negligible.

*Main effects of taste stimuli*

**Table S1**

| **Stevia - control (Nose clip Off)**  Threshold: p=0.05 FWE corrected | | | | | | | |
| --- | --- | --- | --- | --- | --- | --- | --- |
| **Region** | **x(mm)** | **y(mm)** | **z(mm)** | **Z-score** | **voxels** | **p(FWE-corr)** | **p(FDR-corr)** |
| Precentral gyrus | -35 | -28 | 64 | 6.481 | 196 | < 0.0001 | < 0.0001 |
| Postcentral gyrus | -40 | -21 | 52 | 6.170 |  |  |  |
| Caudate | 13 | 15 | 4 | 6.148 | 152 | < 0.0001 | < 0.0001 |
| Caudate | -8 | 10 | 2 | 5.380 |  |  |  |
| Putamen | -18 | 15 | 0 | 5.073 |  |  |  |
| Insula | -30 | 25 | 7 | 5.992 | 78 | < 0.0001 | < 0.0001 |
| Insula | -32 | 18 | 2 | 5.844 |  |  |  |
| Insula | 30 | 27 | 2 | 5.899 | 72 | < 0.0001 | < 0.0001 |
| Insula | 35 | 18 | 7 | 5.128 |  |  |  |
| Sup Motor Area | -4 | 15 | 45 | 5.632 | 26 | < 0.0001 | =0.000623 |
| Sup Motor Area | 4 | 3 | 60 | 5.371 | 46 | < 0.0001 | < 0.0001 |
| Threshold: 0.0001 uncorrected | | | | | | | |
| **Region** | **x(mm)** | **y(mm)** | **z(mm)** | **Z-score** | **voxels** | **p(FWE-corr)** | **p(FDR-corr)** |
| Precuneus | -8 | -69 | 43 | 4.902 | 45 | =0.004325 | =0.018615 |
| Rolandic Operculum | 40 | -4 | 12 | 4.729 | 27 | =0.02498 | =0.067898 |
| Sup Temporal gyrus | -35 | -38 | 7 | 4.717 | 30 | =0.018268 | =0.056552 |
| Precentral gyrus | 32 | -6 | 50 | 4.424 | 38 | =0.008281 | =0.02976 |
| Sup frontal gyrus | 23 | -11 | 55 | 3.968 |  |  |  |

**Table S2**

| **Stevia - control (Nose clip On)**  Threshold: p=0.05 FWE corrected | | | | | | | |
| --- | --- | --- | --- | --- | --- | --- | --- |
| **Region** | **x(mm)** | **y(mm)** | **z(mm)** | **Z-score** | **voxels** | **p(FWE-corr)** | **p(FDR-corr)** |
| Postcentral gyrus | -47 | -18 | 52 | 5.748 | 122 | < 0.0001 | < 0.0001 |
| Precentral gyrus | -37 | -26 | 60 | 5.665 |  |  |  |
| Sup Motor Area | -6 | 13 | 50 | 5.502 | 29 | < 0.0001 | =0.00430 |
| Sup Motor Area | 4 | 15 | 48 | 4.743 |  |  |  |
| Caudate | 1 | 10 | 4 | 5.250 | 10 | =0.00140 | =0.08882 |
| Sup Motor Area | -8 | -6 | 62 | 5.210 | 9 | =0.00178 | =0.09070 |
| Caudate | -16 | 30 | 0 | 5.175 | 11 | =0.00110 | =0.08882 |
| Threshold: 0.0001 uncorrected | | | | | | | |
| **Region** | **x(mm)** | **y(mm)** | **z(mm)** | **Z-score** | **voxels** | **p(FWE-corr)** | **p(FDR-corr)** |
| Caudate | 1 | 10 | 4 | 5.250 | 104 | =0.00014 | =0.00077 |
| Caudate | 8 | 15 | 4 | 4.466 |  |  |  |
| Inferior Frontal Oper | -47 | 10 | 9 | 5.139 | 168 | < 0.0001 | < 0.0001 |
| Insula | -32 | 25 | 7 | 4.848 |  |  |  |
| Insula | 30 | 27 | 7 | 4.918 | 68 | =0.00141 | =0.0052 |
| Mid Temporal gyrus | -37 | -47 | 0 | 4.668 | 24 | =0.04702 | =0.0819 |
| Sup Temporal gyrus | 40 | -45 | 4 | 4.635 | 55 | =0.00357 | =0.0099 |
| Inferior parietal gyrus | -30 | -50 | 43 | 4.526 | 58 | =0.00287 | =0.00908 |
| Sup frontal gyrus | -23 | -4 | 52 | 4.471 | 29 | =0.02956 | =0.06036 |
| Sup parietal gyrus | -20 | -62 | 43 | 4.351 | 29 | =0.02956 | =0.06036 |

abbreviations: Sup, superior; Oper, operculum; mid, middle.
